# Supplementary material for: Antitubercular Activity of 7-Methyljuglone-Loaded Poly-(Lactide Co-Glycolide) Nanoparticles
Source: Pharmaceutics. 2024 Nov 20;16(11):1477. doi: 10.3390/pharmaceutics16111477 (PMC11597334; doi:10.3390/pharmaceutics16111477)
Supplement: Supplementary file 1 [file pharmaceutics-16-01477-s001.zip › pharmaceutics-3279303-supplementary.pdf]

Supplementary materials

Synthesis of the PLGA nanoparticles

Figure S1-S3 illustrates the synthesis of the blank PLGA nanoparticles (Figure S1), the 7-MJ nanoparticles (Figure S2) and the 7-MJ + RhB nanoparticles (Figure S3)

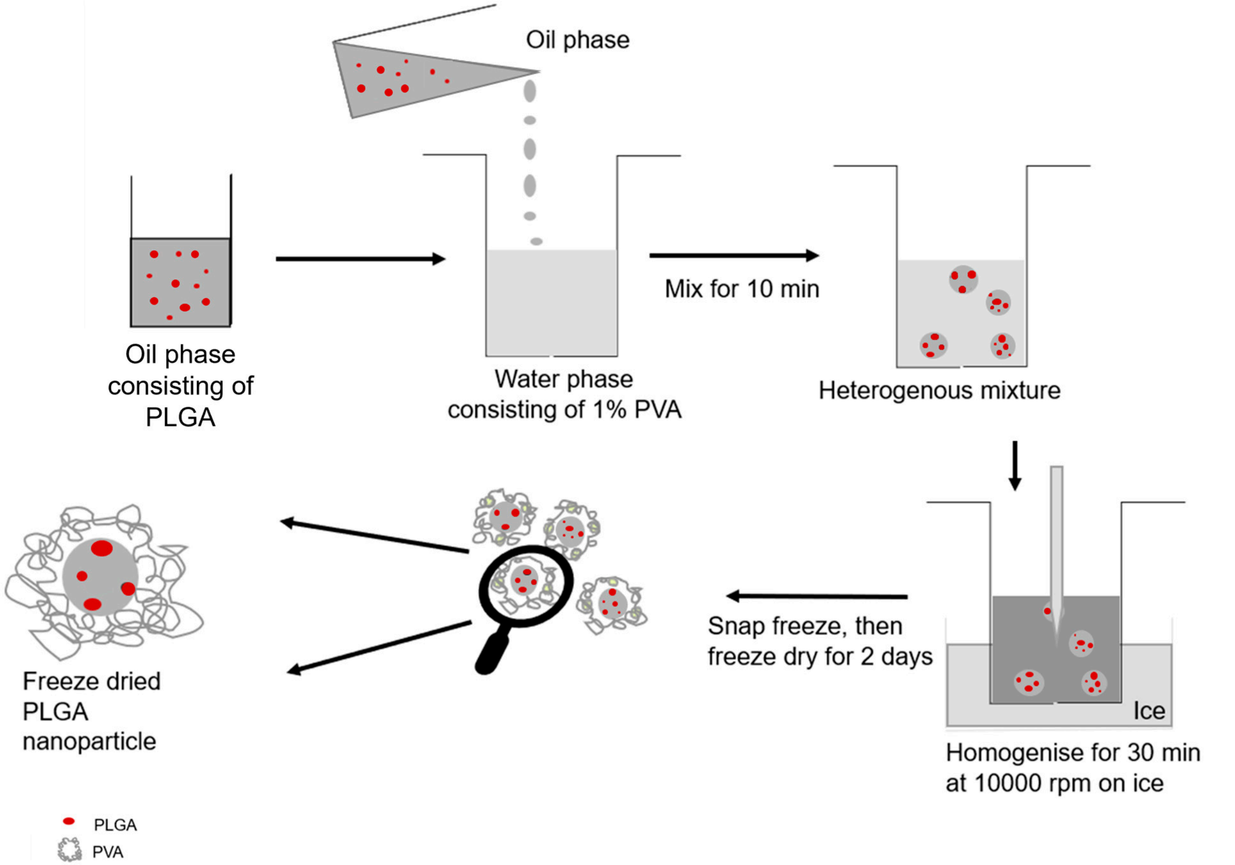

Figure S1: Illustrative diagram of the blank PLGA nanoparticle formulation process.

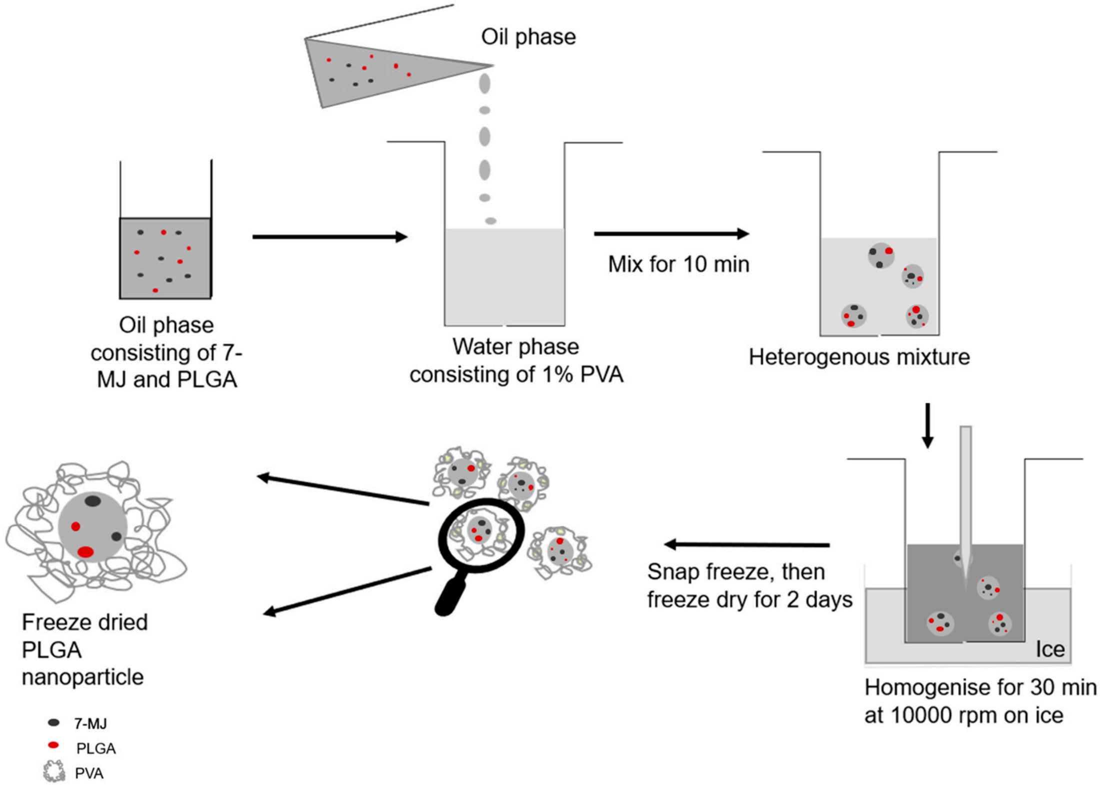

Figure S2: Illustrative diagram of the 7-MJ PLGA nanoparticle formulation process.

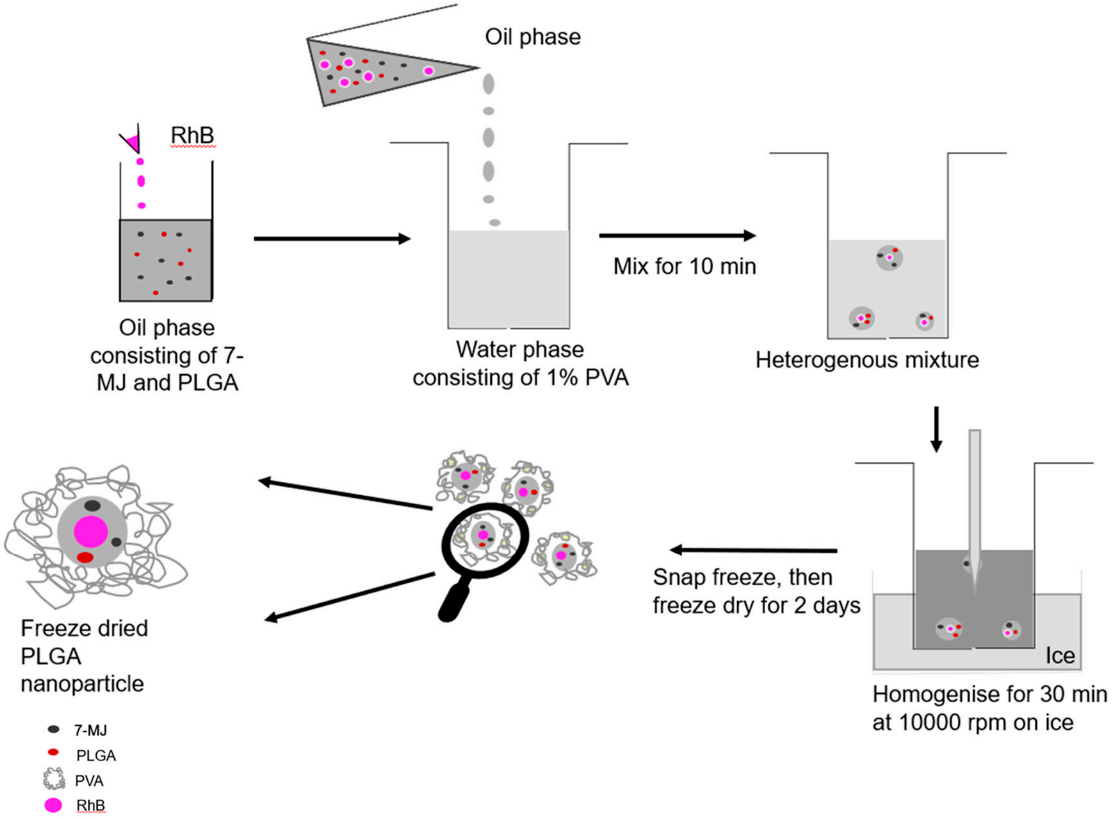

Figure S3: Illustrative diagram of the 7-MJ+RhB PLGA nanoparticle formulation process.

Stability of the PLGA nanoparticles

Storage-stability studies

The storage-stability of the PLGA nanoparticles stored at -20 ˚C, was evaluated over a 6-month period (Table S1). The mean hydrodynamic diameter of the blank nanoparticles, 7-MJ nanoparticles, and 7-MJ+RhB nanoparticles were 260.51 ± 4.95 nm, 274.37 ± 8.31 nm, and 649.94 ± 33.52 nm, respectively over the 6 months. The average PdI of the blank nanoparticles and the 7-MJ nanoparticles were 0.10 ± 0.02 and 0.10 ± 0.03 respectively, whereas the 7-MJ+RhB nanoparticles had an average PdI of 0.55 ± 0.05, over the 6 months. The nanoparticles showed a pH of around ± 8. Over the 6-months the zeta potential of the nanoparticles stayed relatively similar with some fluctuations and decrease over time.

Table S1: The storage stability of the blank nanoparticles, 7-MJ nanoparticles and 7-MJ+RhB nanoparticles stored at -20 ˚C over a course of 6 months.

Month(s)	Mean hydrodynamic diameter (nm)	PdI[a]	Zeta (ζ) Potential (mV)	pH
**Blank nanoparticles**				
0	257.10 ± 2.97	0.10 ± 0.009	- 14.27 ± 0.76	8.33
1	253.60 ± 7.13	0.11 ± 0.03	- 12.50 ± 0.30	8.33
2	255.83 ± 1.23	0.11 ± 0.07	- 17.40 ± 0.30	8.18
3	262.13 ± 6.83	0.05 ± 0.03	- 10.61 ± 0.69	8.38
4	265.87 ± 2.59	0.11 ± 0.02	- 11.79 ± 0.44	8.89
5	265.47 ± 4.04	0.11 ± 0.08	- 13.10 ± 2.03	8.83
6	263.6 ± 4.58	0.12 ± 0.02	- 11.20 ± 1.05	8.80
Average	260.51 ± 4.95	0.10 ± 0.02	- 12.98 ± 2.30	8.53 ± 0.29
**7-MJ nanoparticles**				
0	288.27 ± 4.76	0.09 ± 0.03	-14.00 ± 0.15	8.06
1	265.47 ± 7.51	0.12 ± 0.04	- 12.97 ± 0.46	8.03
2	267.60 ±2.98	0.07 ± 0.01	- 17.57 ± 0.67	8.03
3	269.77 ± 1.82	0.06 ± 0.02	- 17.97 ± 0.60	8.09
4	282.63 ± 6.31	0.13 ± 0.03	- 12.28 ± 1.59	8.02
5	275.03 ±2.22	0.11 ± 0.01	- 10.81 ± 0.19	8.26
6	271. 83 ± 2.89	0.09 ± 0.006	- 11.07 ± 0.88	8.19
Average	274.37 ± 8.31	0.10 ± 0.03	- 13.81 ± 2.92	8.10 ± 0.09
**7-MJ+RhB nanoparticles**				
0	667.30 ± 15.70	0.59 ± 0.03	- 20.53 ± 0.45	7.94
1	678.53 ± 10.63	0.55 ± 0.02	- 17.07 ± 0.84	7.96
2	672.20 ± 14.14	0.61 ± 0.04	- 17.47 ± 0.76	7.97
3	685.5 ± 0.51	0.45 ± 0.11	- 18.63 ± 0.40	8.02
4	626.90 ± 14.99	0.56 ± 0.02	- 19.40 ± 0.61	7.97
5	615.5 ± 5.14	0.55 ± 0.02	- 19.14 ± 0.56	7.88
6	603.63 ± 13.90	0.55 ± 0.03	- 19.45 ± 0.76	7.95
Average	649.94 ± 33.52	0.55 ± 0.05	- 18.81 ± 1.20	7.96 ± 0.04

[a] Polydispersity index, Blank: no compound incorporated, 7-MJ: 7-Methyljuglone, RhB: Rhodamine B

In vitro stability in various biological media

The in vitro mean hydrodynamic diameter stability of the PLGA nanoparticles was evaluated in various biological mediums (Figure S4). The blank nanoparticles and the 7-MJ nanoparticles had a slight linear increase in size, however stayed stable over the course of 6 days (144 h) in pH 4, pH 7, phosphate buffered saline (PBS), Roswell Park Memorial Institute (RPMI) 1640 media, RPMI 1640 media media with 0.1 % phorbol 12-myristate 13-acetate (PMA), 0.5 % bovine serum albumin (BSA), 5 % sodium chloride (NaCl), distilled water (dH2O), 7H9 media and 7H9 media with Tween 80. The blank and 7-MJ nanoparticles exhibited poor stability with a pH of 10 (Figure S4 (C)). The size of the blank nanoparticles and the 7-MJ nanoparticles increased exponentially in 0.5 % cysteine (Figure S4 (H)). The stability of the 7MJ + RhB nanoparticles was only tested in PBS, RPMI 1640, RPMI 1640+PMA, 0.5 % NaCl and dH2O (Figure S4 (D, E, F, I, and J)), as it was formulated solely for the engulfment studies to visualize the nanoparticle uptake into cells. There is stability in size from 2 to 72 h in the above-mentioned mediums, except for PBS, with slight fluctuations. In the cytotoxicity analysis as well as the engulfment studies, the cells in media were incubated for 72 h after treatment with the 7MJ + RhB nanoparticles, therefore the nanoparticles were stable for the duration of the experiments.

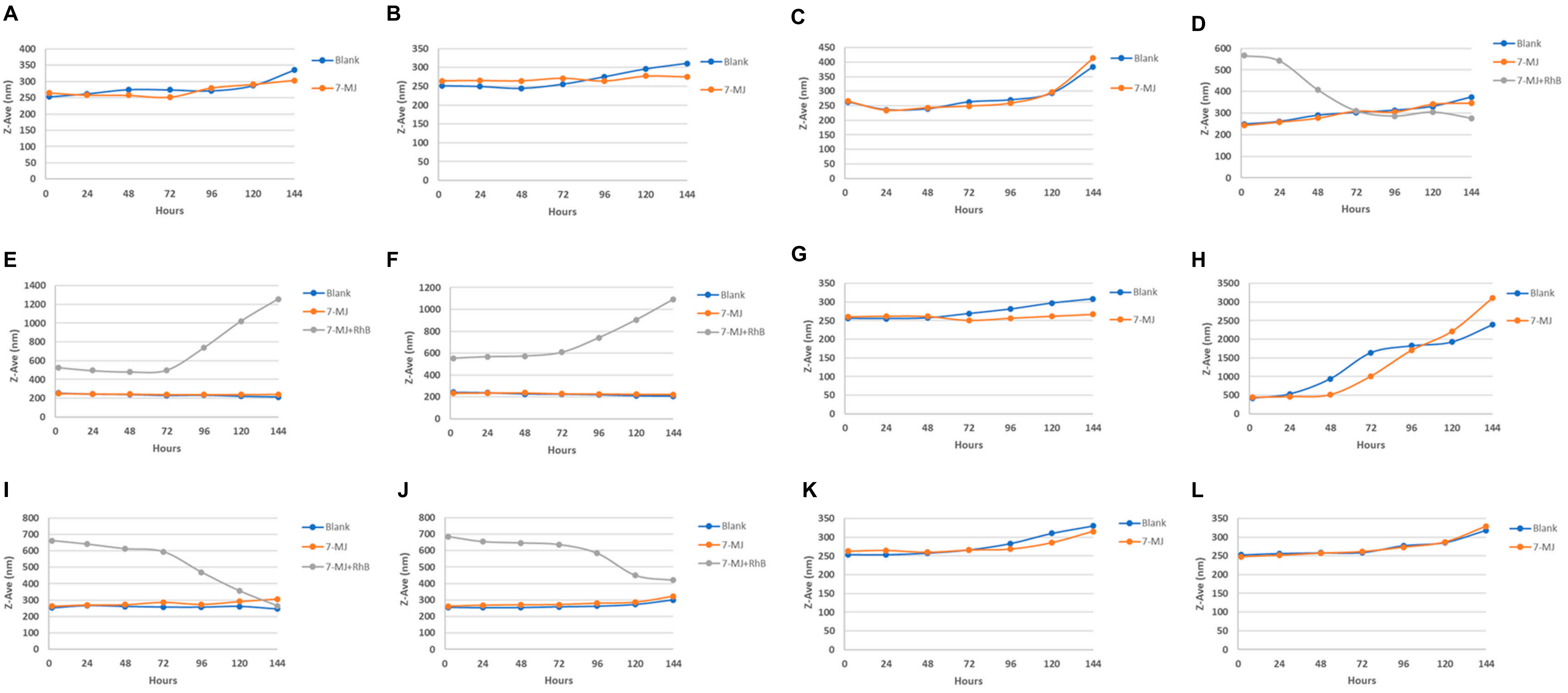

Figure S4: In vitro mean hydrodynamic diameter (Z-Ave) stability of the formulated nanoparticles in (A) pH 4, (B) pH 7, (C) pH 10, (D) PBS, (E) RPMI 1640, (F) RPMI 1640+PMA, (G) 0.5 % BSA, (H) 0.5 % Cysteine, (I) 5 % NaCl, (J) dH_2O, (K) 7H9 media (L) 7H9 media with Tween 80 over a course of 6 days (144 h).
